# Predicting DNA-binding locations and orientation on proteins using knowledge-based learning of geometric properties

**DOI:** 10.1186/1477-5956-9-S1-S11

**Published:** 2011-10-14

**Authors:** Chien-Chih Wang, Chien-Yu Chen

**Affiliations:** 1 Department of Bio-Industrial Mechatronics Engineering, National Taiwan University, Taipei 106, Taiwan; 2 Department of Computer Science and Information Engineering, National Taiwan University, Taipei 106, Taiwan

## Abstract

**Background:**

DNA-binding proteins perform their functions through specific or non-specific sequence recognition. Although many sequence- or structure-based approaches have been proposed to identify DNA-binding residues on proteins or protein-binding sites on DNA sequences with satisfied performance, it remains a challenging task to unveil the exact mechanism of protein-DNA interactions without crystal complex structures. Without information from complexes, the linkages between DNA-binding proteins and their binding sites on DNA are still missing.

**Methods:**

While it is still difficult to acquire co-crystallized structures in an efficient way, this study proposes a knowledge-based learning method to effectively predict DNA orientation and base locations around the protein’s DNA-binding sites when given a protein structure. First, the functionally important residues of a query protein are predicted by a sequential pattern mining tool. After that, surface residues falling in the predicted functional regions are determined based on the given structure. These residues are then clustered based on their spatial coordinates and the resultant clusters are ranked by a proposed DNA-binding propensity function. Clusters with high DNA-binding propensities are treated as DNA-binding units (DBUs) and each DBU is analyzed by principal component analysis (PCA) to predict potential orientation of DNA grooves. More specifically, the proposed method is developed to predict the direction of the tangent line to the helix curve of the DNA groove where a DBU is going to bind.

**Results:**

This paper proposes a knowledge-based learning procedure to determine the spatial location of the DNA groove with respect to the query protein structure by considering geometric propensity between protein side chains and DNA bases. The 11 test cases used in this study reveal that the location and orientation of the DNA groove around a selected DBU can be predicted with satisfied errors.

**Conclusions:**

This study presents a method to predict the location and orientation of DNA grooves with respect to the structure of a DNA-binding protein. The test cases shown in this study reveal the possibility of imaging protein-DNA binding conformation before co-crystallized structure can be determined. How the proposed method can be incorporated with existing protein-DNA docking tools to study protein-DNA interactions deserve further studies in the near future.

## Background

Gene regulation in organisms relies on specific protein-DNA recognitions in a correct way. Recently, many computational methods have been proposed to predict binding sites on both proteins and DNA [1,2]. Sequence-based approaches employ machine learning approaches and training data from structure database to predict DNA-binding sites on proteins [3-5]. On the other hand, pattern mining or multiple sequence alignment techniques are usually incorporated with large-scale molecular binding information such as chromatin immunoprecipitation (ChIP) experiments to discover protein-binding sites on DNA sequences [6-8].

In recent years, many experimentally determined protein structure models are extensively studied to understand and decipher the binding mechanisms of protein-DNA interactions [9]. With protein-DNA complexes, structure-based algorithms [10-12] construct consensus or profiles of binding sites to complement the sequence-based approaches for identifying transcription factor binding sites. We also have many structure-based methods for predicting DNA-binding sites on proteins using both sequence and structure information [13-15]. Although many methods have been proposed to predict protein-DNA interactions, it remains a challenging task to unveil the exact binding conformation of protein-DNA interactions without crystal complexes.

In addition to *de novo* prediction methods, researchers previously applied structure alignment on a query protein against existing protein-DNA complexes for predicting binding sites and constructing potential binding models [16]. Another way to generate protein-DNA complexes for a query sequence is using homology modelling [17]. Sequence alignment is performed on the query protein and its homologous sequences with complex structures. The advantage of using this approach is no protein structure is required for the query protein in advance. Furthermore, with unbound protein structure available, docking programs [18-20] can be employed to predict the binding locations and orientation between proteins and DNA molecules. Protein-DNA docking is capable to generate novel complexes, which is in particularly useful for the query protein that is not similar to any protein chains in the complex database. However, the predicting accuracy of molecular docking still largely relies on computing resources and the prior knowledge about DNA sequence and conformation.

It has been shown in a recent study that the directionality of normal vectors on protein surface is correlated with that of DNA axes [21]. In other words, it has potential to investigate the DNA-binding location and orientation on protein structures even when protein-DNA complexes are not available. This observation motivates the current study. We first characterize geometric property between protein side chains and DNA bases according to a set of existing protein-DNA complexes. Then, several learning algorithms are employed to analyze the query structure and provide prediction of DNA-binding locations and orientation. More specifically, the proposed method is developed to predict the direction of the tangent line to the helix curve of the DNA groove where the DNA-binding protein is going to bind. The predicted information can be used as the initial guess of docking tools or serve as supplementary information to improve the prediction accuracy of docking results.

## Methods

When given the structure of a query protein, the proposed method first identifies a subgroup of conserved residues that form a compact cluster in space and are categorized to have high DNA-binding propensity. The discovered set of residues is considered as a basic DNA-binding unit (DBU) which is assumed to protrude into DNA grooves, no matter major or minor, for recognizing DNA sequences. To predict the DNA-binding orientation of a local region of the protein-DNA binding interface, we apply principal component analysis (PCA) on some particularly selected atom coordinates in a DBU, in order to determine the direction of the tangent line to the helix curve of the DNA groove bound by the DBU. With the detected DBU, we construct the distribution of each base type around the DBU based on a pre-calculated knowledgebase of 80 geometric models. In the following subsections, we describe each procedure of the proposed method in details.

### Collecting training and testing data

The training data used for constructing the knowledgebase was prepared by referring to [16]. This dataset was collected based on the July 2007 release of Protein Data Bank (PDB) database [22], containing only X-ray structures of protein-DNA complexes with resolution better than 3.0 Å. Protein sequence shorter than 40 amino acids were excluded. The DNA molecule must contain at least six base pairs. It is also required that the protein chain in the complex must have at least five DNA-binding residues (distance to DNA atoms < 4.5 Å). Furthermore, member redundancy is removed by performing sequence alignment, resulting in 179 DNA-binding domains, belonging to 170 PDB files. We name it as the dataset PDB170.

Since the proposed method is a knowledge-based approach, it is important to have an independent test set in which the redundancy between training data and testing data has been carefully eliminated. For this purpose, a set of 11 PDB files of DNA-protein complexes (PDB11) were collected as the testing data by the following procedures. First, 1267 protein-DNA complex structures were collected from PDB (release on May 2009), after removing redundancy by excluding sequences with an identity value greater than 90% against a previously selected sequence. All the 1267 protein-DNA complex structures are with resolution better than 3.0Å solved by X-ray diffraction. Second, we performed BLAST on each chain of the 1267 protein chains against the protein chains in PDB170, and excluded any protein chains with e-value<0.001 or identity>25% against the training protein chains to remove the redundancy between the training data and the testing data. Afterward, the selected chains were clustered by CD-HIT [23] to further remove redundancy within the testing data. Finally only the PDB files with exactly two twisted DNA strands were selected. It is noted that PDB files with unwound DNA positions were also excluded.

### Constructing knowledgebase of geometric propensity between side chains and bases

A knowledgebase of geometric properties was built by recording all the geometric relationships between amino acid-base pairs observed in the training data PDB170. Three types of information were retrieved from the training data. First, we calculated the DNA-binding propensity scores for each amino acid using the following equation:(1)

, where the symbol # is short for the word ‘number’.

Next, we investigate DNA-binding propensity for each atom in amino acids based on the similar idea. We want to know which atom of an amino acid is most likely to interact with DNA bases. We use PDB170 to count the number of bases for each atom of amino acids which are falling within the distance of 4 Å. The top-3 atoms for each amino acid are then considered as the reference frame of each amino acid, which will be used later to align the amino acids of the same type from different structure files when constructing geometric models.

**Figure 1:**
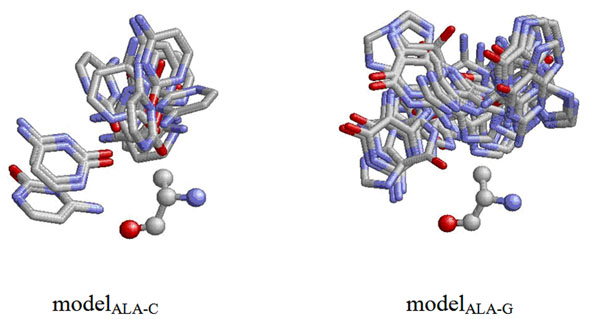
Examples of the geometric models.

Next, we constructed 80 geometric models *M_a_*_-_*_b_* based on PDB170, where *a* is an amino acid type and *b* is a base type. In each model, we collected all the residues of the same type from different structure files and superimposed them by aligning their reference coordinate frame constructed by the three atoms described in the previous paragraph. When performing superimposition, the base atoms belonging to base type *b* falling within the distance of 4Å with respect to the residue were translated and rotated in the same way. In other words, a geometric model stores all the transformed coordinates of the atoms of a particular base type with respect to a particular type of amino acid. Two examples of the 80 models are shown in Figure 1.

### Discovering basic DNA-binding units

A basic DNA-binding unit (DBU) is defined as a compact cluster of residues that is supposed to protrude into DNA grooves when a protein binds to DNA. The proposed method discovers DBUs by combining information of conservation, solvent accessibility, and DNA-binding propensity. Conserved residues are discovered by a pattern mining utility, MAGIIC-PRO [24]. Solvent accessibility of each residue was calculated by DSSP [25]. Finally, conserved residues near surface were clustered based on their spatial relationships, and the resultant clusters were ranked by their DNA-binding propensities. The details of the three procedures are given below.

MAGIIC-PRO is a sequential pattern mining utility which is useful in identifying functional regions and residues [26]. The readers can refer to the paper of MAGIIC-PRO for more details about the parameter settings. After a set of conserved residues were discovered, we calculated the relative solvent accessibility (RSA) score of each residue on the structure of the target protein chain by invoking DSSP [25]. Afterward, we only picked up residues with RSA scores higher than 0.25 for the following clustering process.

Hierarchical clustering was employed to cluster these functionally important surface residues into DBUs. At first, clustering was conducted at atom level. Euclidean distance was used to measure the dissimilarity between two atoms and average linkage was adopted as the scenario to measure dissimilarity between existing clusters. The clustering process was stopped once any pair of cluster exhibit dissimilarity larger than 11 Å (covering about three successive bases in DNA grooves). Once it happens that not all the atoms of a single residue are falling into the same cluster, a majority vote was used to determine the belonging of the residues to clusters. Finally, we used the DNA-binding propensity scores of the clusters to rank them. The score of a cluster is the lumped sum of the DNA-binding propensity score defined in **Eq. (1)** of the residues inside it. The cluster scores higher than expectation (‘number of residues inside the cluster’ × ‘average of DNA-binding propensity of the 20 amino acids’) are considered as DNA-binding units in the following analyses.

### Predicting DNA-binding orientation

The proposed method assumes that the discovered DBUs will protrude into DNA grooves, no matter with major grooves or minor grooves. In this regard, we selected three atoms in amino acids to represent the spatial property of each residue, and use PCA to predict the direction of the tangent line to the helix curve of DNA grooves. The first selected atom is the atom with the highest DNA-binding propensity in an amino acid. The second and the third atoms selected to represent the amino acid are the CA and C atoms on the backbone.

### Predicting locations of DNA bases

Given a predicted DBU and the 80 geometric models of *M_a_*_-_*_b_* in our knowledgebase, the distribution of a particular base type *b* around the DBU in space is estimated as:(2)

, where **x** is a 3-dimensional vector, representing the coordinates of a point in space, **y***_i_* presents the 3-dimensional coordinates of the atom *i*, *aa*(*r*) stands for the amino acid type of the residue *r*, and *C* is the set of residues in the selected DBU. In this study, we empirically used the number of residues belonging to the amino acid type aa(*r*) in the DBU to normalize the contributed scores before accumulating them.

**Figure 2:**
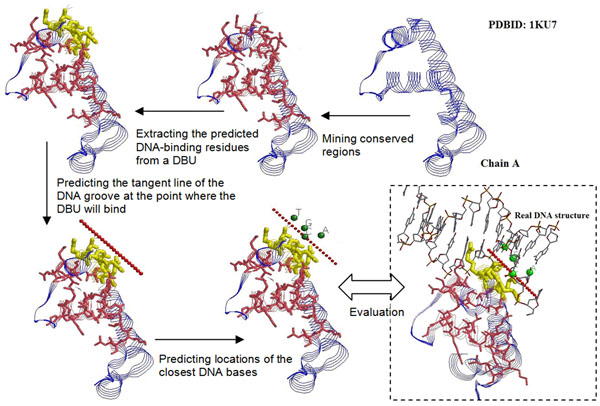
**Procedure flow of the proposed method.** The red sticks are the conserved residues discovered by MAGIIC-PRO and the yellow sticks are the predicted DNA-binding residues from the top-1 DBU.

After the probability estimating where a base is likely to present is modelled, the next step is to find the positions with the maximum probability efficiently. In this study, we used all the base positions collected in the models as the sampling space. This space was reduced first by removing potential coordinates falling in protein cores [27]. The possibility of a base atom falling in the core of the protein was modelled by the following equation:(3)

, where **x***_ij_* is the 3-dimentaional coordinates of the atom *i* in the base *j* and **y***_k_* presents the 3-dimensional coordinates of the atom *k* in the set of CA atoms of the query protein *P*. These *P_core_*(**x***_ij_*) scores were sorted in descending order, and we only kept the bases with low scores until we first reached the condition where the distance between the base and protein is <2Å. Second, the sampling space was further reduced by an incremental clustering procedure. The root mean square deviation (RMSD) values for any pairs of bases in the same model were calculated in advance. Then, the bases were examined according to the scores of **Eq. (2)** in descending order. A base was selected if all of its RMSD values to the previously selected bases are >5. Finally, we reported the position with the highest score in **Eq. (2)** as the predicted center location of DNA bases that will be bound by a DBU. In Figure 2, we use an example to summarize all the procedures of the proposed method.

## Results

In this section, we first define how the performance of the proposed method was evaluated. After that, we demonstrate that how the proposed method can be used to predict DNA-binding conformation for a large DNA-binding interface. A TATA-box binding protein was used as an example in this situation.

### Evaluation of prediction accuracy

**Figure 3:**
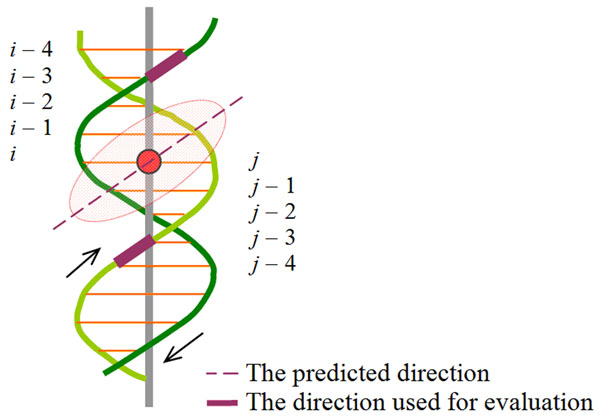
**Demonstration of how the predicted orientation is evaluated.** The red circle stands for the center of a DBU-binding region. In this case, the DBU (the shaded ellipse) protrudes into a major groove.

To evaluate the accuracy of predicting the direction of the tangent line to the helix curve of the DNA groove at the point where a DBU binds, we first record the center base pair identification *i* (or the identification *j* on the other strand) of the binding region of a DBU. After that, we use direction constructed by the two adjacent phosphorous atoms on the backbone of the nucleic acids *i* – 3 and *i* – 4 (or *j* – 3 and *j* – 4) as the putative correct direction (the better one is reported), as exemplified in Figure 3. For minor grooves, of which the size is smaller, the direction constructed by two adjacent phosphorous atoms of the nucleic acids *i* and *i* + 1 (or *j* and *j* + 1 on the other strand) is used instead.

The results were summarized in Table 1. We first examined whether the top-1 DBU identifies a correct binding location. The sampled positions with the highest probability given by **Eq. (2)** are compared to the closest base in the validation complex structure and the distance between the predicted position and the centroid of the closest base is reported. Among the 11 tested cases, four cases are categorized as failures (distance >6Å). One of the failures is owing to the miss of MAGIIC-PRO in identifying correct functional regions. The other three failures were due to mis-predictions of the proposed method to identify correct DBUs. For the seven successful cases in detecting DBUs, PCA correctly constructs the direction of the DNA grooves on five cases (the cosine value of two direction vectors is larger than 0.9, i.e. angle <25°). This reveals the possibility of predicting protein-DNA binding orientation without co-crystallized structures.

**Table 1:** Errors of the predicted locations and orientation on the 11 test cases (PDB11).

PDB ID and the chain ID	Location error in Å	Groove type to which the top-1 DBU binds	Orientation error in degree
1KU7:A	0.8	Major	7.7
1RIO:A	3.6	Major	21.3
*2R1J:L*	*8.7*	*NA*	*NA*
2Z3X:A	4.0	Minor	5.0
1A3Q:A	5.7	Major	19.6
3DFX:A	2.4	Major	10.7
2O49:A	*8.8*	*NA*	*NA*
*2E1C:A*	*23.2*	*NA*	*NA*
3ERE:D	2.7	Major	41.9
*1BDT:A*	*13.6*	*NA*	*NA*
3CLC:A	5.1	Major	63.7

The four cases for which the proposed method failed to find a correct binding location are shown in italic form.

### Multiple predictions for large protein-DNA interfaces

**Figure 4:**
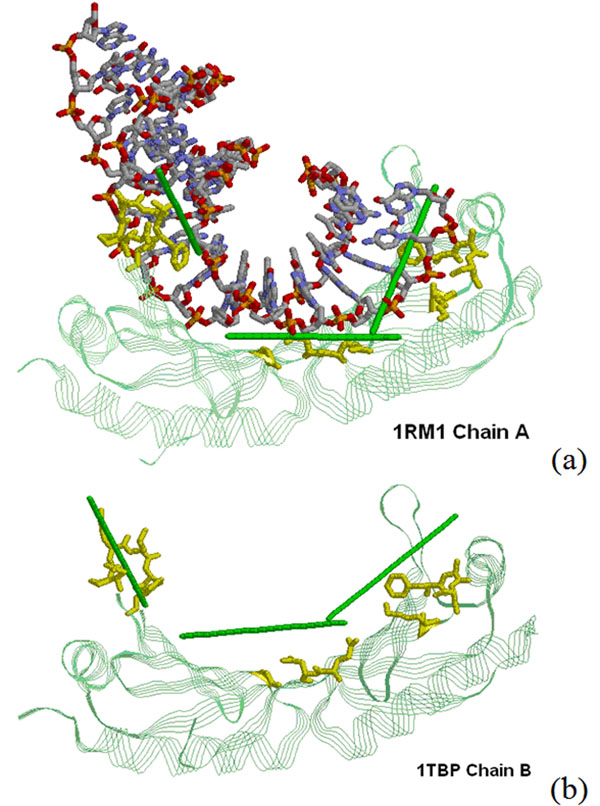
**Multiple location and orientation predictions** (a) Predictions on three DBUs for 1RM1:A. (b) Predictions on unbound structure (1TBP:B). In both figures, the protein is shown in green using *Strands* presentation. Residues in one DBU are shown in yellow using *Sticks* presentation. The predicted tangent lines of the DNA groove for three DBUs are plotted as green sticks, and the real DNA structure is presented in CPK color using *Sticks* presentation.

If the protein-DNA interacting interface is large or when DNA is considerably bended, it is needed to repeat the proposed procedure of predicting DNA-binding locations and orientation on a few of the top-ranked clusters. We use the TATA-box binding protein of *Saccharomyces cerevisiae* (1RM1:A) as an example to illustrate the basic idea. As shown in Figure 4(a), all the top three clusters are shown to close to DNA. It is also shown in Figure 4(a) that our predictions correctly predict the route of DNA molecules when compared to the real DNA structure that was superimposed into the figure for visualization.

### Prediction using unbound protein structure

Since proteins usually undergo conformation change upon binding DNA, it is of interest to investigate that how the proposed method performs when the given protein structure is an unbound model. We use another structure model (1TBP:B) for the same protein (TATA-box binding protein of *Saccharomyces cerevisiae*) to predict DNA-binding locations and orientation by the proposed method. In Figure 4(b), we show that the predictions are generally consistent with that derived from the bound structure shown in Figure 4(a). This reveals the potential of the proposed method in future applications of predicting exact binding mechanisms using unbound structures of DNA-binding proteins alone.

## Discussion

**Figure 5:**
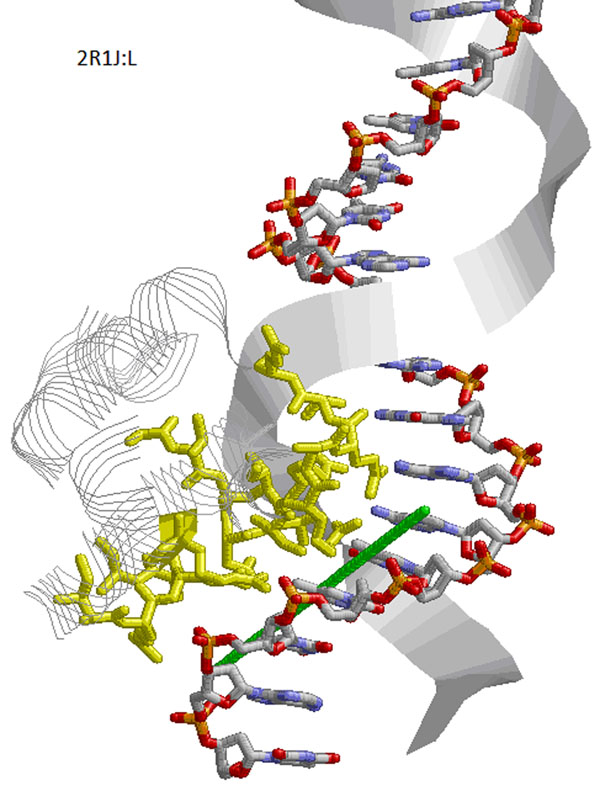
**Orientation prediction based on the DNA-binding residues predicted by DISPLAR** The query protein (2R1J:L) is shown in grey using *Strands* presentation. The predicted DNA-binding residues are shown in yellow using *Sticks* presentation. The predicted tangent direction of the DNA groove is plotted using a green stick, and the real DNA structure is presented in CPK color, with one strand in *Sticks* presentation and the other strand in *Ribbons* presentation.

The four failures on location prediction in Table 1 implied that some of the predicted DBUs are not actually close to DNA. It was suspected that the proposed method might not be good enough in predicting DNA-binding residues, which might further degrade the performance of the proposed method on orientation prediction. To clarify this point, we collected the set of surface conserved residues discovered by MAGIIC-PRO followed by RSA screening for each test case, and compared them to the DNA-binding residues predicted by a structure-based approach (DISPLAR [14]) and a sequence-based approach (NAPS [5]) proposed recently. The results of the analysis is provided in Table 2, where the precision rate is defined by the number of true positives (TPs) divided by the number of predicted residues, the sensitivity rate is defined by the number of TPs divided by the number of real contact residues (distance to DNA <4.5Å), and a TP is a correct prediction on a contact residue. It is shown in Table 2 that the structure-based approach (DISPLAR) is generally more capable than the proposed method in delivering a good-quality set of residues for DBU prediction, though it fails to make any predictions on two cases (2Z3X:A and 1BDT:A). An example shown in Figure 5 demonstrates that the PCA analysis conducted on the DNA-binding residues predicted by DISPALR successfully depicted the direction of the tangent line of the DNA major groove. This suggests the possibility of improving the proposed method by incorporating structure-based DNA-binding residue predictors in the near future.

**Table 2:** Comparison with existing methods for predicting DNA-binding residues.

		Precision				Sensitivity	
			
PDB ID: chain ID	NAPS	DISPLAR	The proposed method		NAPS	DISPLAR	The proposed method
1KU7:A	0.27	0.55	**0.56**		0.57	**0.79**	0.71
1RIO:A	0.20	**0.81**	0.25		0.40	**0.65**	0.30
*2R1J:L*	0.48	**0.84**	0.39		0.62	**0.76**	0.43
2Z3X:A	0.35	0.00 ^a^	**0.48**		0.38	0.00	**0.57**
1A3Q:A	0.08	**0.47**	0.30		**0.53**	**0.53**	0.47
3DFX:A	0.24	0.43	**0.55**		0.45	**0.65**	0.30
2O49:A	0.15	0.38	**0.43**		0.36	0.36	**0.86**
*2E1C:A*	0.09	**0.88**	0.06		0.46	**0.54**	0.15
3ERE:D	0.16	**0.67**	0.35		0.50	**0.80**	0.30
*1BDT:A*	**0.36**	0.00 ^a^	0.30		**0.57**	0.00	**0.57**
3CLC:A	0.38	**0.91**	0.75		0.56	**0.63**	0.38

Average	0.25	0.54	0.40		0.49	0.52	0.46

The residues discovered by MAGIIC-PRO and with RSA>25% are collected as the prediction set of the proposed method for comparison. The best records among the three methods are bolded for both precision and sensitivity rates in each row.

^a^ no DNA-binding residues were predicted.

## Conclusions

This study opens an opportunity of computational methods to imagine protein-DNA binding conformation as long as protein structures are available. Using MAGIIC-PRO to discover functionally important residues achieves 10 successes among the 11 test cases. The proposed method for discovering basic DNA-binding units achieves seven successes among the 10 good cases from MAGIIC-PRO. Among the seven correctly predicted DBUs, the constructed models identify correct base locations for all the cases and the PCA analysis successfully identify the tangent direction of the bound groove on five cases. We concluded that the proposed method could help to set the initial conditions of DNA structure models for conducting protein-DNA docking or serve as useful supplementary information in studying protein-DNA interactions.

## Competing interests

The authors declare that they have no competing interests.

## Authors' contributions

CYC initiated the study. CCW and CYC developed the methods together. CCW implemented all the program codes and performed the analyses. CCW and CYC interpreted the results and wrote the manuscript together. Both authors read and approved the final manuscript.
